# Structure-Activity Relationships for Some Diamine, Triamine and Schiff Base Derivatives and Their Copper(II) Complexes

**DOI:** 10.1155/MBD.1998.323

**Published:** 1998

**Authors:** C. A. Bolos, G. St. Nikolov, L. Ekateriniadou, A. Kortsaris, D. A. Kyriakidis

**Affiliations:** 1 Laboratory of Inorganic Chemistry Dept. of Chemistry Aristotle University of Thessaloniki 54006 Greece; 2 Department of Physical and Theoretical Chemistry University of Plovdiv Plovdiv 4000 Bulgaria; 3 Institute of General and Inorganic Chemistry Bulgaan Academy of Sciences Sofia 1113 Bulgaria; 4 Laboratory of Biochemisty Dept. of Chemistry Aristotle University of Thessaloniki 54006 Greece; 5 Theagenion Cancer Institute Thessaloniki 54007 Greece

## Abstract

Ethylenediamine (en), putrescine (pu), diethylenetriamine (dien), dipropylenetriamine (dpta), spermidine (spmd) and their Cu^II^ compounds as well as the Schiff bases with 2-furaldehyde (dienOO), 2- thiophenecarboxaldehyde (dienSS) and pyrrole-2-carboxaldehyde (dienNN) of dien and that of dpta with 2- thiophenecarboxaldehyde (dptaSS), were prepared and characterised. They were tested against *Bacillus substilis*, *Bacillus cereus*, *Staphylococcus aureus*, *Escherichia coli*, *Proteus vulgaris* and *Xanthomonas campestris* as antibacterial reagents, the highest activity being exhibited by Cu(dptaSS)(NO_3_)_2_ complex, which acts as antibiotic. In the antiproliferative tests (vs. T_47_D,L_929_ and BHK_21/c13_ cell lines) the best results were obtained with Cu(dptaSS)^2+^ and Cu(dienSS)^2+^. Electronic structure calculations gave for dptaSS and dienSS the higher negative charges on the N atoms. The counter-ions (Br^-^, NO^3^_-_ and SO^4^_2-_) play an important role by modulating the reagent's selectivity versus the bacteria [Gram(+) or Gram(-)], but they have no effect on the antiproliferative activity.


### STRUCTURE-ACTIVITY RELATIONSHIPS FOR SOME DIAMINE, TRIAMINE AND SCHIFF BASE DERIVATIVES AND THEIR COPPER(II) COMPLEXES

C. A. Bolos*[1], G. St. Nikolov*[2,3] L. Ekateriniadou[4], A. Kortsaris[5] and D. A. Kyriakidis[4]

[1] Laboratory of Inorganic Chemistry, Dept. of Chemistry, Aristotle University of Thessaloniki, 54006, Greece

[2] Department of Physical and Theoretical Chemistry, University of Plovdiv, Plovdiv 4000, Bulgaria

[3] Institute of General and Inorganic Chemistry, Bulgarian Academy of Sciences, Sofia 1113, Bulgaria

[4] Laboratory of Biochemistry, Dept. of Chemistry, Aristotle University of Thessaloniki, 54006, Greece

[5] Theagenion Cancer Institute, Thessaloniki 54007, Greece

**Abstract.** Ethylenediamine (en), putrescine (pu), diethylenetriamine (dien), dipropylenetriamine (dpta), spermidine (spmd) and their $Cu^{II}$ compounds as well as the Schiff bases with 2-furaldehyde (dienOO), 2-thiophenecarboxaldehyde (dienSS) and pyrrole-2-carboxaldehyde (dienNN) of dien and that of dpta with 2-thiophenecarboxaldehyde (dptaSS), were prepared and characterised. They were tested against *Bacillus substilis*, *Bacillus cereus*, *Staphylococcus aureus*, *Escherichia coli*, *Proteus vulgaris* and *Xanthomonas campestris* as antibacterial reagents, the highest activity being exhibited by $Cu(dptaSS)(NO_3)_2$ complex, which acts as antibiotic. In the antiproliferative tests (vs. $T_{47}D$, $L_{929}$ and $BHK_{21/c13}$ cell lines) the best results were obtained with $Cu(dptaSS)^{2+}$ and $Cu(dienSS)^{2+}$. Electronic structure calculations gave for dptaSS and dienSS the higher negative charges on the N atoms. The counter-ions ($Br^-$, $NO_3^-$ and $SO_4^{2-}$) play an important role by modulating the reagent's selectivity versus the bacteria [Gram(+) or Gram(-)], but they have no effect on the antiproliferative activity.

### Introduction

Di- and triamines and, in general, polyamines are biologically occurring substances resulting from the metabolism of ornithine, bleomycin, etc. [1]. They are excellent complexating reagents, capable of co-ordinating to a number of transition metal ions, including $Cu^{II}$ [2,3]. Cyclic amines such as imidazole, histamine, etc. and their co-ordination compounds have been extensively studied [2] but the acyclic amines, despite their occurrence as terminal amines in the metabolism of natural products such as bleomycin [4], have received less attention. So far the growth inhibitory activity of the metal- ion complexes has been correlated to the atomic mass [5], electronegativity [6], atomic radii [7], the number of unpaired electrons of the metal ion [8], and, in general, to the electronic structure parameters of the transition metal compounds [9]. Some of these correlations, however have been questioned [2b,9] and are no longer generally accepted (vide infra).

Important physicochemical parameters [10] of biofunctional ligands are their lipid solubility, charge distribution, polarisability and steric parameters. Transition metal compounds with such ligands are very potent chemotherapic reagents, the $Pt^{II}$-compounds taking first place [11]; the $Cu^{II}$ compounds take a lower ranking position, but they are also important because of their "plasticity" [12], i.e. they are capable of assuming different shapes with different co-ordination numbers and thus adapt to the substrate [13].

At least two factors are known [6,10] to be important for the growth inhibitory activity of co-ordination compounds: (a) the ease to adopt certain geometry and thus avoid possible steric hindrance during their physiological action; (b) the partition coefficient between lipid and water media; it depends strongly on the charges of the atoms in the active molecule. Ligands, co-ordinated to a metal ion, can modify both factors and thus enhance or lower their growth inhibitory properties. Thus, for example, a ligand can reduce the charge on the metal ion through electron donation and in this way it can ease the permeability of the metal ion into the cell. Electrostatic interaction of a positively charged complex with adjacent negative groups of the biomolecules can confer rigidity to the fluxional ionic adducts which result from such an interaction and modify their biological function.

In this paper we report the synthesis, characterisation and bacterial growth inhibitory properties of some acyclic di- and triamines and their Schiff base derivatives as well as their $Cu^{II}$ complexes. An attempt has been made also to trace their antiproliferative activity and to relate their inhibitory and cytostatic properties with their electronic structure parameters.

The following ligands and their $Cu^{II}$ compounds have been studied:

| | |
|---|---|
| Ethylenediamine (en) | $H_2NCH_2CH_2NH_2$ |
| Putrescine (pu) | $H_2N(CH_2)_4NH_2$ |
| Diethylenetriamine (dien) | $H_2N(CH_2)_2NH(CH_2)_2NH_2$ |
| Dipropylenetriamine (dpta) | $H_2N(CH_2)_3NH(CH_2)_3NH_2$ |
| Spermidine (spmd) | $H_2N(CH_2)_4NH(CH_2)_3NH_2$ |

Schiff bases of dien with 2-furaldehyde (dienOO), 2-thiophene-carboxaldehyde (dienSS) and pyrrol-2-carboxaldehyde(dienNN) and their $Cu^{II}$ compounds as well as a Schiff Base of dpta with 2-thiophene-carboxaldehyde (dptaSS) and its $Cu^{II}$ compound were also prepared. Some of the $Cu^{II}$ compounds have been isolated with different counter-ions ($Br^-$, $NO_3^-$ and $SO_4^{2-}$).

Several of our ligands and their $Cu^{II}$ compounds have been studied in solution as to the possible equilibria and stability [14-16]. For the pu complex several species were found, CuHL, $CuL_2(OH)^+$, and at pH 9 precipitation is known to occur [16].

In the above selection we have two diamines (en and pu) with chains of different length, and three triamines (dien, dpta and spmd), two of which are symmetric (dien and dpta) with chains of different length, and one of them is asymmetric (spmd). This choice offers a chance to study the effect of number of bonding N atoms, length of aliphatic chain, symmetry of the two branches of chains and the modifying effect of the N-substituents.

The compounds were characterised by their IR and electronic spectra, magnetic moments and elemental analyses.

The geometric structures were determined by Molecular Mechanics (MMP2) [17-21], which can take into account the $\pi$-electronic conjugation in some of the ligands. The MM2 input parameters were used and those of Cu should be specifically mentioned: the bond stretching force constants Cu-N were set to be 0.89 mdyn $Å^{-1}$. Bending force constants NCuN were set to 0.25 mdyn $rad^{-1}$ for the angles N-Cu-N=90.0 deg, and 0.35 mdyn $rad^{-1}$ for the remaining angles. The torsional parameters CCNCu were set to zero, since they had little effect on the energy values. These constants compare well with those obtained from normal co-ordinate analysis for similar Cu-compounds [22]. The molecular structures obtained were compared with available crystal structures [23-27]. The electronic structures of the free ligands were examined by the Austin Model 1 (AM1), which is a version of the MNDO method [28]. The parameters used were those included in the MOPAC 6.0 database. The electronic structures of the $Cu^{II}$ compounds were examined by the Extended Huckel method [29], since no parameters for copper are available for the AM1 method. The parameters used in the EH calculations were those of Murphy and Fitzpatrick [30]. Although the EH results should be viewed with caution as to their absolute values, they are fully credible used in comparing a series of similarly constituted compounds [29b].

### Experimental Part

### Preparation of ligands and chelates

The ligands en, pu, dpta and spmd were commercially available. The dien and dpta Schiff base ligands were prepared by the condensation reaction of the corresponding carboxaldehydes with dien taken in molar ratio 2:1. The compounds $Cu(dien)(NO_3)_2$ and $Cu(dienSS)(NO_3)_2$ were prepared and characterized as described elsewhere [25,31]. The compounds $Cu(dienOO)(NO_3)_2$, $Cu(dienNN)(NO_3)_2$, $Cu(dienSS)Br_2$, $Cu(dienSS)SO_4$, $Cu(dptaSS)(NO_3)_2$ were prepared according to the scheme 1.

2-Thiophenecarboxaldehyde or pyrrole-2-carboxaldehyde or 2-furaldehyde (20 mmol) were mixed with 10 mmol diethylenetriamine or dipropylenetriamine. The resulting product was dissolved in methanol (50 ml) and $Cu(NO_3)_2.3H_2O$, $CuSO_4.5H_2O$ or $CuBr_2$ in methanol (20 ml) were added. The mixture was stirred for an hour and after 12 h of staying the precipitates were isolated by filtration, washed successively with methanol and ether, and dried in vacuum. The compounds were re-crystallised from methanol. They represent uniform blue crystals or blue crystalline powders with different colours. Analytical results together with the measured magnetic moments and electronic spectra are given in Table I.

The compounds $Cu(en)_2(NO_3)_2$, $Cu(pu)_2(NO_3)_2$, $Cu(spmd)(NO_3)_2$ were prepared by evaporation of a $Cu(NO_3)_2.3H_2O$ solution in water (50 ml) of the appropriate amine amount as described elsewhere [30].

### Characterisation of the $Cu^{II}$ Compounds

Elemental analysis. - The blue micro-crystalline complexes are stable in air. Elemental analyses gave 1:1 Cu-L stoichiometry, where L is the polyamine ligand. With $NO_3^-$, $SO_4^{2-}$ or $Br^-$ completing the square planar co-ordination sphere, this indicates 4-co-ordinated $Cu^{II}$. Data on C, H, N and Cu contents are given in Table I. The C, H, N were analysed with a Perkin-Elmer elemental analyser. Copper was determined by atomic absorption spectroscopy.

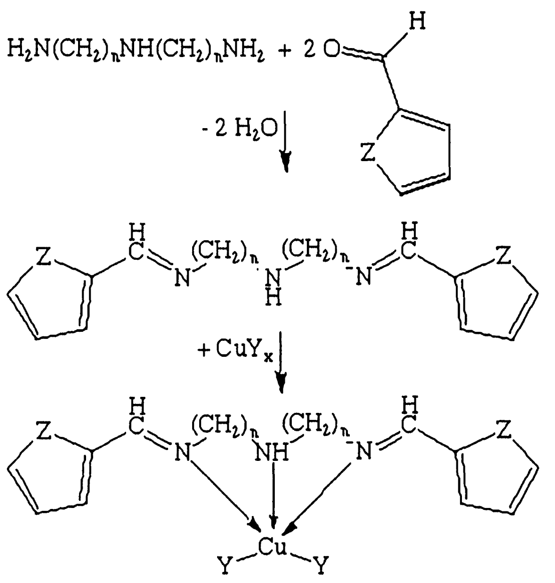

$Z = S, O$ or NH; $n = 2$ or 3; $x = 1$ or 2; $Y = Br, NO_3$ or $2Y = SO_4$

Scheme 1

Solubility. - The complexes are soluble in water, methanol and in co-ordinating solvents such as DMF and DMSO. This a strong indication that the compounds are ionic.

Conductivity. - Molar conductivities were measured with a WTW conductivity bridge and a calibrated dip-type cell. The molar conductivity of the aqueous solutions was 110-138 $\mu S\ cm^{-1}$ which is within the range of 1:1 electrolyte [32] (vide infra, the discussion of the IR spectra).

Electronic spectra. - Electronic spectra (Table I) were recorded on a Shimadzu UV 160A spectrophotometer in the 200-800 nm region using aqueous solutions of the compound. The electronic spectra of the $Cu^{II}$ complexes in aqueous solutions exhibit absorption bands at 610-639 nm and 247-276 nm with another band at 295-298 nm for IV, VI and VII. The 615-639 nm band is of low intensity and is assigned to a d-d transition in 4-co-ordinate $Cu^{II}$, while the 247-276 nm band should be assigned either to a $\pi \rightarrow \pi^*$ interligand transition in the Schiff base ligands or to $L \rightarrow M$ charge transfer transition (vide infra, the electronic structure calculations). The 295-298 nm band is not related to the nature of the ligand or counter ion and should be assigned to $\pi \rightarrow \pi^*$ interligand transition.

Magnetic moments. - Magnetic susceptibilities of powdered samples were recorded at 25 °C by the Faraday method with a home-made balance against $Hg[Co(SCN)_4]$ as calibrant. Diamagnetic corrections were estimated from Pascal constants. The measured magnetic moments (Table I) were 1.73 BM for I-IV and 1.83 BM for VII, which is consistent with monomeric structures; 1.61 and 1.66 BM were recorded for V and VI, respectively, possibly indicating the presence of metal-metal interaction through magnetic exchange coupling in polymeric structures.

Infrared spectra. -IR spectra were recorded with a Perkin-Elmer 1640 FT-IR spectrophotometer in the 200-4000 $cm^{-1}$ region using KBr pellets (Table II). They show some bands that are typical of diethylenetriamine or dipropylenetriamine, respectively [22]. The 1665 $cm^{-1}$ band in the free dien ligand spectrum was assigned to stretching the C=N bond in the -CH=N-groups. This band is shifted to lower frequencies (about 55-60 $cm^{-1}$) upon co-ordination, proving that the -CH=N- group nitrogen atoms are co-ordinated to $Cu^{II}$.

In the $Cu^{II}$ nitrate compounds the 1740 and 1760 $cm^{-1}$ bands prove the presence of uncoordinated nitrate groups while the 1380 and 1350 $cm^{-1}$ bands indicate a second unidentate nitrate group. These results are in agreement with molar conductivity measurements which show that the complexes behave as 1:1 electrolytes in solution dissociating according to the scheme:

$$[Cu(L)(NO_3)_2] \rightarrow [Cu(L)(NO_3)]^+ + NO_3^-.$$

Hence the IR spectra, the magnetic and molar susceptibility measurements suggest that $Cu^{II}$ in the CuL-nitrate complexes (L is a tridentate ligand) is in a 4-co-ordinate $CuN_3O$ chromophore.

For the $Cu(dienSS)(SO_4)$ complex the 1145, 1120, 1045, 1030 and 960 $cm^{-1}$ bands can be assigned to a bridging sulphate group.

Table I. Analytical Data, Electronic and Magnetic Data of the $Cu^{II}$ Compounds

| No | Compound | %C Found (Calc.) | %H Found (Calc.) | %N Found (Calc.) | %Cu Found (Calc.) | UV-VIS nm (log ε) | $\mu_{eff}$ (BM) |
|---|---|---|---|---|---|---|---|
| I | $Cu(dien)(NO_3)_2$ | 16.45 (16.52) | 4.41 (4.47) | 23.95 (24.09) | 21.60 (21.87) | 615(2.11) 251(3.08) | 1.73 |
| II | $Cu(dienSS)(NO_3)_2$ | 35.06 (35.11) | 3.53 (3.55) | 14.57 (14.63) | 13.21 (13.28) | 634(2.1) 270(4.1) | 1.73 |
| III | $Cu(dienOO)(NO_3)_2$ | 37.53 (37.62) | 3.75 (3.81) | 15.60 (15.68) | 14.02 (14.22) | 639(2.2) 276(4.73) | 1.73 |
| IV | $Cu(dienNN)(NO_3)_2$ | 37.51 (37.79) | 4.23 (4.27) | 21.90 (21.92) | 14.07 (14.29) | 610(2.33) 298(4.19) 247(3.98) | 1.73 |
| V | $Cu(dienSS)Br_2$ | 32.53 (32.67) | 3.21 (3.31) | 8.06 (8.17) | 12.14 (12.35) | 637(2.22) 261(4.26) | 1.61 |
| VI | $Cu(dienSS)(SO_4$ | 37.21 (37.29) | 3.70 (3.77) | 9.21 (9.19) | 14.00 (14.10) | 634(2.16) 295(4.45) 271(4.41) | 1.66 |
| VII | $Cu(dptaSS)(NO_3)_2$ | 37.75 (37.90) | 4.10 (4.15) | 13.67 (13.82) | 12.45 (12.54) | 624(2.18) 295sh 264(4.28) | 1.83 |

Table II. The most important IR bands (in $cm^{-1}$) of the studied $Cu^{II}$ Compounds

| No | Compound | $\nu_{as}$(N-H) | $\nu_s$(N-H) | ν(C=N) | ν($NO_3$) | ν($NO_3$) | ν(Cu-N) | ν(Cu-O) |
|---|---|---|---|---|---|---|---|---|
| I | $Cu(dien)(NO_3)_2$ | 3280 | 3220 3140 | | 1755 1735 | 1375 1310 | 520 | 430 |
| II | $Cu(dienSS)(NO_3)_2$ | | 3130 | 1635 | 1760 1745 | 1380 1335 | 520 | 430 |
| III | $Cu(dienOO)(NO_3)_2$ | | 3230 3120 | 1640 | 1760 1745 | 1390 1340 | 515 | 465 |
| IV | $Cu(dienNN)(NO_3)_2$ | 3280 | 3230 3150 | 1635 | 1760 1740 | 1380 1350 | 530 | 470 |
| V | $Cu(dienSS)(Br)_2$ | 3390 | 3220 3100 | 1605 | --- | ---- | 515 | ---- |
| VI | $[Cu(dienSS)(SO_4)]$ | | 3210 3070 | 1605 | 1145[a] 1120[a] | 1045 1030[a] 960[a] | 520 | 425[b] |
| VII | $Cu(dptaSS)(NO_3)_2$ | 3300 | 3240 3180 | 1610 | 1760 1745 | 1380 1360 | 520 | 450 |

[a] These are $SO_4^{2-}$ bands: (1145,1120 (doublet), 1045,1030 (doublet), 960cm$^{-1}$).

[b] Cu-O from the $SO_4^{2-}$ group

The ν(Cu-N) band is spread over three peaks in the 515-530 $cm^{-1}$ range which indicates that the Cu-N bonds are slightly different in a single compound but do not differ much in the different compounds. The data for compounds I and VI prove that the counter-ion has little effect on the Cu-N(dien) bands and the same holds for II and V, for which substituting $NO_3^-$ with $Br^-$ brings about a decrease in ν(Cu-N) of only 5 $cm^{-1}$. Compounds with the same counter ion ($NO_3^-$) form the series:

compound IV > II = I > III = V

ν(Cu-N) 530 520 515 $cm^{-1}$

The bands ν(Cu-O) (O from $NO_3$) vary in the 430-470 $cm^{-1}$ range - much wider than for the ν(Cu-N) band. It also indicates a greater differences in the Cu-O bonds in the different compounds.

The IR spectra of the free Schiff-base ligands and those of the respective $Cu^{II}$ compounds were (within experimental error) the same in the regions where the 5-membered $C_4H_4O$, $C_4H_4NH$ and $C_4H_4S$ rings absorb, thus proving that these substituents do not participate in bonding to $Cu^{II}$.

**Biological Tests**

**Antibacterial Activity**

The antibacterial activity (Table III) of all the studied compounds has been evaluated against *Bacillus substilis* (ATTCC 6633), *Bacillus cereus* (ATTCC 11778), *Staphylococcus aureus*, *Escherichia coli* (XL 1), *Proteus Vulgaris* and *Xanthomonas campestis* (ATTCC 13951). The screening was performed by the Minimal Inhibitory Concentration (MIC) [33]. Two different media were used. Mueller Hinton Broth (MHB, Institute Pasteur # 64887) and Minimal Salts Broth (MSB, 1.5% glucose, 0.5 % $K_2HPO_4$, 0.2% $NH_4Cl$, 0.1% NaCl, 0.01% $MgSO_4$ and 0.1% yeast extract, pH 7). The compounds were dissolved in distilled water with a 2-fold successive serial dilution from 800 to 25 μg $ml^{-1}$. All cultures were incubated at 28 °C. Control tests with no active ingredients were also carried out.

Table III. Bacterial Screening Data by the MIC (in μg $ml^{-1}$ ) method[a]

| No | Compound | B. cereus | S. aureus | E. coli | X. campestris |
|---|---|---|---|---|---|
| | | Gram(+) | Gram(+) | Gram(-) | Gram(-) |
| I | $Cu(dien)(NO_3)_2$ | | | | 200(800)[b] |
| II | $Cu(dienSS)(NO_3)_2$ | | 800 | | 200(800)[b] |
| III | $Cu(dienOO)(NO_3)_2$ | | | | 400 |
| IV | $Cu(dienNN)(NO_3)_2$ | | 400 | | 400(800)[b] |
| V | $Cu(dienSS)(Br)_2$ | | | | 400(800)[b] |
| VI | $Cu(dienSS)(SO_4)$ | | 400(800)[b] | | 400(800)[b] |
| VII | $Cu(dpta)(NO_3)_2$ | 400 | 400 | 400 | |
| VIII | $Cu(dptaSS) (NO_3)_2$ | 100(800)[b] | | | |
| IX | $Cu(en)_2(NO_3)_2$ | | | 800 | |
| X | $Cu(Pu)_2(NO_3)_2$ | 800 | 800 | 800 | |
| XI | $Cu(Spmd) (NO_3)_2$ | 800 | 800 | 800 | |
| XII | Dien | | 400 | 800 | 400 |
| XIII | DienSS | | 800 | | 800 |
| XIII | En | | | 400 | |
| XIV | DptaSS | | 800 | | |

The compound $Cu(NO_3)_2.3H_2O$, $CuSO_4.5H_2O$, $CuBr_2$ as well as the ligands dienOO, dienNN, putrescine(Pu), spermidine(Spmd) and dipropylenetriamine(dpta) showed no antibacterial action. All ligands and Cu compounds are inactive against *B.substilis* and *Pr.vulgaris.*

[a] Averages from 3 dilutions.

[b] The data in parentheses refer to cultures in Mueller Hinton broth.

[c] Blanks indicate no action for concentrations lower than 800 μg $ml^{-1}$;

800-400 μg $ml^{-1}$ indicate weak activity, 200 μg $ml^{-1}$ indicate satisfactory activity and 100 μg $ml^{-1}$ indicate strong activity.

Several findings emerge from Table III.

a/ Both ligands and complexes were inactive against *B.Substilis* (Gram +) and *Pr. Vulgaris* (Gram -). For this reason they were omitted in Table III.

b/ The ligands pu, spmd, dpta, unlike their $Cu^{II}$ complexes were inactive against all studied bacteria.

c/ While there are marked differences in the bacterial screening tests in MSB with different $Cu^{II}$ compounds, the results from tests in MHB indicate very poor performance in all tests (>800 μg $ml^{-1}$). This can be attributed to the different compositions of the two broths (vide infra).

d/ The best results were obtained with $Cu(dptaSS)(NO_3)_2$ (100 μg $ml^{-1}$) against *B. cereus* (Gram +). It is highly selective, since it is inactive against all the other bacteria. Next come $Cu(dien)(NO_3)_2$ and $Cu(dienSS)(NO_3)_2$ against *X. campestris* and their action is also highly selective.

e/ Moderate success (400 μg $ml^{-1}$) is achieved by $Cu(dienOO)(NO_3)_2$ against *X. Campestris*, and by $Cu(dienNN)(NO_3)_2$ against *S. aureus* and *X. campestris*; $Cu(dienSS)(Br)_2$ acts selectively against *X. campestris* and $Cu(dienSS)(SO_4)$ against *S.aureus* and *X. campestris.*

It can be thus concluded that the number of N atoms (en vs. dien), length of chain (dien vs. dpta) of the polyamines is essential for their antibacterial activity: compounds of tridentate ligands with longer aliphatic chains perform better than compounds of bidentate ligands with shorter chains. The N-substituents in the Schiff bases (dien vs. dienSS, dienOO and dienNN), however, seem to be highly effective since the $Cu(dienSS)^{2+}$- and $Cu(dptaSS)^{2+}$-species are particularly active.

There are significant differences in activity between the copper complex and the corresponding ligand and that differences are highly selective with respect to the studied bacteria. We take a few salient examples:

With respect to *X. campestris*:

dien (400 μg $ml^{-1}$) vs. Cu(dien) $(NO_3)_2$ (200 μg $ml^{-1}$); dienSS (800 μg $ml^{-1}$) vs. Cu(dienSS) $(NO_3)_2$ (200 μg $ml^{-1}$).

With respect to *B. cereus*:

dptaSS (800 μg $ml^{-1}$) vs. Cu(dptaSS) $(NO_3)_2$ (100 μg $ml^{-1}$).

In fact the last example shows that the ligand dptaSS is weakly active, while its complex acts as an antibiotic.

The two broth media contain different constituents: MHB contains peptone and starch and MSB contains glucose and phosphate as main constituents. The latter may bind to $Cu^{II}$ and invoke replacement of the ligands of the original complex if the stability constants of $Cu^{II}$ with glucose and phosphate are greater than those with the polyamine ligands. Were this the case, all complexes should have the same activity in a given broth. This is exactly the case with MHB but not with MSB. Hence, it may be concluded that the peptone and/or the starch form more stable complexes with $Cu^{II}$, replacing the polyamine ligands. The opposite case holds for the MS broth since presumably the glucose and phosphate complexes of $Cu^{II}$ are less stable than the corresponding polyamine complexes.

**Cell Culture and Antiproliferative Activity**

$T_{47}D$ cells from metastatic pleural effusion of patients were grown in Dulbecco medium plus 10% fetal bovine serum. Mouse fibroblast cells $L_{929}$ and Baby Hamster Kidney fibroblast were grown in Eagle's Minimal Essential Medium plus 10% fetal bovine serum $BHK_{21/c13}$. Antiproliferative activity (Table IV) was evaluated in cells grown on a monolayer. The number of cells was measured by the Trypan Blue method [34]. The ligands show no activity while the complexes behave as growth inhibitors with $ID_{50}$ values ranging from 60 to 250 μg $ml^{-1}$ .

Table IV. Antiproliferative Activity ($ID_{50}$ μg $ml^{-1}$) of $Cu^{II}$ Compounds

| No | Compound[a] | $BHK_{21/c13}$ cells | $L_{929}$ cells | $T_{47}D$ cells |
|---|---|---|---|---|
| I | $Cu(dien)(NO_3)_2$ | 250 | 230 | 210 |
| II | $Cu(dienSS)(NO_3)_2$ | 75 | 85 | 68 |
| III | $Cu(dienOO)(NO_3)_2$ | 180 | 200 | 180 |
| IV | $Cu(dienNN)(NO_3)_2$ | 170 | 180 | 160 |
| V | $Cu(dienSS)(Br)_2$ | 80 | 90 | 75 |
| VI | $Cu(dienSS)(SO_4)$ | 75 | 80 | 72 |
| VII | $Cu(dptaSS)(NO_3)_2$ | 65 | 65 | 60 |

[a] Ligands have no effect.

[b] The data in this Table are averages of three measurements; statistics gave ± 2-3 $ID_{50}$ values.

A survey of Table IV shows the following trends:

a/ All compounds perform slightly better against $T_{47}D$ cells than against BHK and slightly worse against $L_{929}$ cells.

b/ Differences of up to 3 times in the $ID_{50}$ values are evident for the different compounds against each cell line. The activity of the compounds can be categorised in the following series:

VII > VI = II > V >> IV, III > I for $BHK_{21/c13}$;

VII > VI > II > V >> IV > III > I for $L_{929}$;

VII > II > VI ~V >> IV > III > I for $T_{47}D$.

The seven compounds, however, form two separate groups:

group A (II,VI,VII,V) with 60- 90 $ID_{50}$ values; group B (IV,III,I) with 160-250 $ID_{50}$ values.

Group A contains the three Cu(dienSS)-compounds with different counter-ions, while the best performer in this group is the outsider $Cu(dptaSS)^{2+}$-complex. It is thus evident that the counter-ion plays a very modest role and the active species in the first group are $Cu(dptaSS)^{2+}$ and $Cu(dienSS)^{2+}$, both containing the 2-thiophenecarboxaldehyde substituent, $Cu(dptaSS)^{2+}$ doing slightly better than $Cu(dienSS)^{2+}$.

The reason for the large differences in activity of the $Cu^{II}$ Schiff-base compounds with different substituents will be examined further by calculations on the molecular and electronic structures which may shed light on the problem.

**Molecular and Electronic Structures of the Ligands and the $Cu^{II}$ Compounds**

Both the ligands (en, pu, spmd) and their $Cu^{II}$ complexes have been extensively studied (see, for example, the collection in ref. [35]). In our tests the best results were obtained with dpta and dien and Schiff-base dien and dpta and for this reason we shall focus our discussion on the molecular and electronic structures of these ligands and their $Cu^{II}$ complexes. The $Cu^{II}$ dien [23] and dpta [24] complexes were studied before, but their Schiff-base substituted complexes were not.

(a) Comparison of the Results for the Dien, DienOO, DienNN, DienSS and DptaSS Ligands

It is seen from the $\Delta H_f$ values in Table V that dpta is the most stable ligand and, among the dien derivatives, dien itself is the most stable one. DienSS ranks last in stability.

Here and everywhere else $N^A$ refers to N from the -CH=N-C- (N is $sp^2$ hybridised) or $-NH_2$ (N is $sp^3$ hybridised) groups; $N^I$ refers to N from the central C-NH-C group (N is $sp^3$ hybridised). With the exception of dienSS, MM and AM1 gave practically the same values 3.02-3.98 Å for the $N^A$-$N^I$ distance in dien and substututed diens with no dependence on the substituents. In dpta and dptaSS, the $N^AN^I$ distance depends on the substituent: 3.1 and 3.4 Å, respectively. The $N^A$-$N^A$ distances show a very complicated picture: 5.1 Å for dien, 4.6-4.9 Å for substituted diens; 4.3 Å for dpta and finally 4.4 Å for dptaSS. These values probably reflect both the difference in chain length and the repulsion between the bulky N-substituents.

Table V. Molecular Structure of the Dien, Dpta and Dien and Dpta Schiff Base Ligands in ωShape

| Compound | | $\Delta H_f$ kcal $mol^{-1}$ | SE kcal $mol^{-1}$ | $N^AN^I$ Å | $N^AN^A$ Å | $N^AN^IN^A$ degree |
|---|---|---|---|---|---|---|
| Dien | MM | -5.4 | 0.24 | 2.93 | 5.14 | 122.2 |
| | AM1 | -16.5 | | 3.02 | 5.43 | 128.1 |
| DienSS | MM | 147.8 | 22.42 | 2.86 | 4.59 | 109.8 |
| | AM1 | 96.7 | | 3.02 | 5.17 | 117.7 |
| DienOO | MM | 73.6 | 29.03 | 2.91 | 4.88 | 113.6 |
| | AM1 | 48.6 | | 3.06 | 5.29 | 119.7 |
| DienNN | MM | 129.3 | 47.76 | 2.89 | 4.72 | 110.0 |
| | AM1 | 117.7 | | 3.05 | 5.24 | 118.4 |
| Dpta | MM | -11.9 | 6.33 | 3.06 | 4.25 | 87.9 |
| | AM1 | -20.8 | | 3.13 | 3.94 | 78.1 |
| DptaSS | MM | 140.8 | 26.90 | 3.42 | 4.37 | 79.3 |
| | AM1 | 85.9 | | 3.48 | 4.40 | 77.0 |

$\Delta H_f$ - heat of formation; SE - strain energy; $N^A$ refers to N from the -CH=N-C or $-NH_2$ group; $N^I$ refers to N from the central C-NH-C group;

The $N^AN^IN^A$ angle is defined by the two $N^AN^I$ lines. The lowest $N^AN^IN^A$ angle is obtained for the dptaSS and dpta ligands (88 and 79 deg), the highest $N^AN^IN^A$ angle for dien (122 deg). These values correlate with the $N^AN^A$ distance: the longer the $N^AN^A$ distance, the larger the $N^AN^IN^A$ angle. Further it might be expected that the smaller the $N^AN^IN^A$ angle, the longer the Cu-$N^I$ bond should be.

By comparing the charges on the nitrogen atoms it is readily seen (Table VI, the second numbers in the rows refer to the ligands) that the charges on $N^I$ are almost the same (-0.33 by AM1), while the charge on $N^A$ shows a great variation when passing from dien to dienSS, dienOO and dienNN (-0.33, -0.18, -0.17, -0.20); the charge on $N^A$ in dien and dpta are practically the same (-0.33 and -0.31, respectively); same trend is observed with dienSS and dptaSS (-0.18, -0.17). The explanation is that $N^I$ is always $sp^3$ hybridised and does not participate in π-conjugation with the $N^A$-substituents, while $N^A$ is also $sp^3$ in un-condensed di- and triamines but $sp^2$ in the Schiff bases, in which electron density is taken away from $N^A$ and localised at the 5-membered rings, dienOO and dienSS doing slightly better than dienNN. There is no such delocalisation for un-condensed dien and dpta and they show the highest charges on both types of nitrogen atoms.

The HOMO values for the four dien ligands are (-9.65, -9.05, -9.07, -8.71, eV for dien, dienSS, dienOO, and dienNN, respectively) suggesting that dienNN is the best electron donor (highest HOMO), while dien is the worst electron donor in this series. The LUMO values (2.88, 0.30, -0.06, -0.28 eV) rank definitely dienNN as the best electron acceptor (lowest LUMO), dien taking up the last (worst) electron accepting position.

The difference LUMO-HOMO for the studied ligands roughly correlates with the first UV bands (compare Tables I and VI) and for this reason these bands may be assigned tentatively to interligand electron transitions, as suggested when discussing the electronic spectra (vide supra).

(b) Comparison of the results for the $Cu^{II}$ Co-ordination Compounds

It is seen from the $\Delta H_f$ results (Table VII) that the most stable species is $Cu(dpta)^{2+}$ ($\Delta H_f$ = -772 kcal $mol^{-1}$) with $Cu(dien)^{2+}$ ($\Delta H_f$ = -716 kcal $mol^{-1}$) coming next and the most unstable is $Cu(dienSS)^{2+}$ ($\Delta H_f$ = -228 kcal $mol^{-1}$).

The variations of the charge on the $Cu^{II}$ atom are (Table VI): 0.170, 0.098, 0.184, 0.186, 0.171, 0.329 for $Cu(dien)^{2+}$, $Cu(dienSS)^{2+}$, $Cu(dienOO)^{2+}$, $Cu(dienNN)^{2+}$, $Cu(dpta)^{2+}$ and $Cu(dptaSS)^{2+}$, respectively. The lowest charge on the Cu atom is in the $Cu(dienSS)^{2+}$ unit and it is almost 2-3 times lower in comparison with the Cu charge in the other units (see Table VI). The high negative charge on $N^A$ indicates that the Cu-N bonds in Cu(dienSS) are the most ionic ones, which favours higher water-lipid partition coefficients and defines a better growth inhibitory activity [6,10]. It should be
noted that there are less electrons on the aldimine nitrogen ($q_N$=0.01) than on the nitrogens which take part in the conjugation with the N-substituents [$q_N$=-0.63 in $Cu(dptaSS)^{2+}$] and this is exactly the opposite as compared with the free ligands - the trend is reversed.

Table VI. Electronic Structure Parameters for Dien, Dpta and Dien and Dpta Schiff Bases (AM1 results) and Their $Cu^{II}$ Dipositive Ions (EH results); HOMO and LUMO in eV.

| | Cu(dien) | Cu(dienSS) | Cu(dienOO) | Cu(dienNN) | Cu(dpta) b | Cu(dptaSS) |
|---|---|---|---|---|---|---|
| $q_{Cu}$ | 0.170/ | 0.098/ | 0.184/ | 0.186/ | 0.171/ | 0.329/ |
| $q_N^A$ | 0.057/-0.332 a | -0.290/-0.179 | -0.128/-0.167 | -0.030/-0.201 | -0.023/-0.31 | -0.632/-0.167 |
| $q_N^I$ | 0.229/-0.309 | -0.019/-0.308 | 0.178/-0.307 | 0.176/-0.306 | 0.167/-0.36 | 0.009/-0.303 |
| HOMO = IP | -11.99/-9.65 | -11.56/-9,05 | -11.86/-9.07 | -11.87/-8.71 | -12.22/-9.06 | -11.06/-9.14 |
| LUMO | -4.95/+2.88 | -8.67/-0.30 | -8.75/-0.06 | -8.62/0.28 | -5.18/3.23 | -10.56/-0.23 |
| LUMO-HOMO | 7.04/12.53 | 2.89/8.75 | 3.11/9.01 | 3.25/8.95 | 7.04/12.29 | 0.50/8.91 |

[a] First number refers to the Cu-compounds, second numbers refers to the ligand.
[b] Data for $Cu(dpta)^{2+}$ taken from ref. [21].

Table VII. Thermodynamic Stability and Molecular Shapes of the $Cu^{II}$ Dien and $Cu^{II}$ Dpta Schiff Base Ions (Charged +2) with Symmetric Ligand Conformations. Results from MM Calculations

| | Cu(dien) | Cu(dienSS) | Cu(dienOO) | Cu(dienNN) | Cu(dpta) | Cu(dptaSS) |
|---|---|---|---|---|---|---|
| $\Delta H_f$ kcal $mol^{-1}$ | -716.36 | -227.92 | -316.79 | -253.86 | -772.55 | -383.35 |
| SE kcal $mol^{-1}$ | -710.81 | -454.65 | -462.72 | -436.79 | -754.82 | -496.21 |
| 2 x $N^AN^I$ Å | 2.68 | 2.60 | 2.62 | 2.62 | 2.92 | 2.76 |
| 1 x $N^AN^A$ Å | 3.67 | 3.90 | 3.89 | 3.89 | 3.51 | 3.63 |
| $N^AN^IN^A$ deg | 86.4 | 97.2 | 95.9 | 95.9 | 73.64 | 82.12 |
| 2 x $CuN^A$ Å | 1.87 | 1.97 | 1.96 | 1.96 | 1.88 | 1.87 |
| 1 x $CuN^I$ Å | 1.87 | 1.87 | 1.87 | 1.87 | 1.80 | 1.80 |
| 2 x $N^ACuN^I$ (º) | 78.9 | 82.2 | 82.9 | 82.9 | 106.4 | 97.5 |
| 1 x $N^ACuN^A$ (º) | 157.8 | 164.5 | 165.8 | 165.8 | 140.0 | 153.8 |

Another trend emerges when comparing the HOMO and LUMO values. It is readily seen that the HOMO values are almost constant (-11.3 to -12.2 eV) while the LUMO values vary much (from -4.95 eV for $Cu(dien)^{2+}$ to -10.56 eV for $Cu(dptaSS)^{2+}$. Thus, all Cu-units may be comparable as to their electron donor abilities, which are expressed in terms of the HOMO values, but they differ widely as to their electron accepting abilities, which are expressed in terms of the LUMO values. The Schiff base complexes are much better electron acceptors (LUMO -8.62 to -10.56 eV than the dien and dpta complexes LUMO -4.95 and -5.15 eV, respectively) and the extraordinary high negative LUMO for $Cu(dptaSS)^{2+}$ should be noted.

The bands in the visible part of the electronic spectrum (610-639 nm) roughly correlate with the LUMO-HOMO difference (compare Tables I and VI), and since they consists mainly of Cu d-AO they may tentatively assigned to d-d transitions (vide supra).

**Correlation between Biological Activity and Electronic Structure**

The results from the antibacterial tests suggest that $Cu(dptaSS)(NO_3)_2$ is the best antibacterial agent against *B. cereus*. $Cu(dien)(NO_3)_2$ and $Cu(dienSS)(NO_3)_2$ act best against *X.campestris*. The antibacterial activity depends on the counter-ion and for this reason it might be suggested that the active species is CuLX, where X is the respective counter-ion included in the first co-ordination sphere.

In the antiproliferative studies, the $Cu(dptaSS)^{2+}$ and $Cu(dienSS)^{2+}$ species have the highest activity, thus emphasising that the presence of 2-thiophenecarboxaldehyde is essential. In the case of $Cu(dienSS)^{2+}$ the counter-ion plays no effect and it may be thus suggested that the species penetrating the cells are CuL, stripped of that ion. The species $Cu(dptaSS)^{2+}$ has the highest positive charge on copper and the highest negative charge on N, which favours its partitioning between the water and lipid phases during the tests. The lowest LUMO makes the species an excellent electron acceptor in further reactions with redox partners. In contrast, the charge on Cu in Cu(dienSS) is the lowest and charges on N are also low. The thermodynamic stability seems to be a minor factor. It may thus be concluded that different factors influence the activities of $Cu(dptaSS)^{2+}$- and $Cu(dienSS)^{2+}$-species. The common feature of the two species is a high HOMO (-11.06 and -11.56 eV, respectively).

**Conclusions**

The $Cu^{II}$ compounds with triamines tested in the present study are almost uniform as to their molecular structures, the predominant group being a Cu atom surrounded by 3N and one X atom in an almost planar arrangement; however, they show subtle variations as to their electronic structures. They have revealed themselves as selective reagents with respect to some Gram-positive and Gram-negative bacteria, the selectivity being modulated by the counter ions such as $NO_3^-$, $SO_4^{2-}$, etc., which can co-ordinate to $Cu^{II}$. The ionicity of the Cu-N bonds in the tested compounds seems to correlate with their growth inhibitory action - the high positive charge on the Cu ion, and the high negative charge on the N atoms improve the antibacterial activity, probably due to better water-lipid partitioning ratios [6,10]. While the species active as bacteriostatic reagents include the counter-ions which complete the co-ordination sphere of $Cu^{II}$ up to 4, the species active as cytostatic reagents seem to be stripped of these counter-ions and may be co-ordinatively unsaturated. The site presented at the $CuN_3$ unit and its electronic and molecular parameters may thus be expected to be the determining factor for the interaction with the cyto-substrates.

Acknowledgement. Financial help through grant #446 of the Bulgarian National Research Fund is gratefully acknowledged. One of us (GSN) thanks the EC Science Directorate for providing help under contract # ERBCIPACT 920337.

**References**

[1] Canellakis E. S., Viceps-Madore D., Kyriakidis D. A. and Heler J. S in *Current Topics in Cellular Regulation,* Horecker B. L. and Stadtman E. R., eds., Acad. Press, New York p. 155 (1979).
[2] (a) Kitajima N., *Adv. Inorg. Chem.*, **29,** 1 (1992) and refs. therein. (b) Farrell, N. *Transition Metal Complexes as Drugs and Chemotherapeutic Agents*, Kluwer, 1989.
[3] Newman E. C. and Frank C. W. *J. Pharm. Sci.* **65,** 7729 (1976).
[4] Umezawa H. and Takita T. *Structure and Bonding*, **4,** 77 (1980) and refs therein.
[5] McCallan A. E. S. and Willcoxon F. *Contributions By the Thompson Institute*, **6,** 479 (1934).
[6] Horsfall J. G. in *Principles of Fungicidal Action Chronica Botanica company*, Waltham M. A., p. 132 (1956).
[7] Srivastava R. S., *Inorg. Chim. Acta*, **55**,: L71 (1981).
[8] Golding M., Lehtonen K. and Ralph B. J., *Austr. J. Chem.*, **28,** 2393 (1975).
[9] Que L. ed. *Metal Clusters in Proteins, ACS Symposium Series* p. 372, ACS Washington, DC.
[10] Keverling J. A. in *Biological Activity and Chemical Structure*, vol. **21** (1977), E. J. Arends ed. Elsevier, Amsterdam p.35.
[11] Lippert J. *Progr Inorg. Chem.*, **3,** 72 (1989).
[12] Gazo J., Bersuker I. B., Garaj J., Kabesova M., Kohent J., Langfelderova H., Melnik M., Serator M. and Valach F., *Coord. Chem. Revs.*, **19,** 253 (1976).
[13] Bacci M. *New J. Chem.*, **17,** 67 (1993).
[14] Lomazik L. and Gasowska A., *Monatshefte fur Chemie,* **124,** 109 (1993).
[15] Lomazik L. and Wojciechowska A., *Polyhedron*, **8,** 2645 (1989).
[16] Wojciechowska A., Bolewski L. and Lomazik L. *Monatshefte fÅr Chemie* **122,** 131 (1991).

[17] (a) Brubaker G. R. and Johnson D. W., *Coord. Chem. Revs.*, **53**, 1 (1984); (b) Hancock R. D., *Progr. Inorg. Chem.*, **37**, 187 (1989); (c)Comba P., *Coord. Chem. Rev.*, **123**, 1 (1993); (d)Hay B. P., *Coord. Chem. Rev.*, **126**, 177 (1993).
[18] Comba P. and Zimmer M., *Inorg. Chem.* **33**, 5368 (1994).
[19] Clack T, *A Handbook of Computational Chemistry.* J Wiley &Sons, New York,. J J.Gajewski and K E. Gilbbert, Indiana University, USA (1985).
[20] Allinger N. L., *J. Am. Chem. Soc.*, **49**, 8127 (1977); the program is distributed by Quantum Chemistry Program Exchange (QCPE) as No. 395, Bloomington, Indiana, USA.
[21] Wu Y. D., Houk K. N., Valentine J. S. and Nam W., *Inorg. Chem.*, **31**, 718 (1992).
[22] Trendafilova N., Nikolov G. St., Bauer G., Kellner R., *Inorg. Chim. Acta*, **210**, 77 (1993) and refs. therein.
[23] Allmann R., Krestl M., Bolos C., Manoussakis G. and Nikolov G. St., *Inorg. Chim. Acta*, **175**, 255 (1990).
[24] Gliemann G., Klement U., Stuckel C., Bolos C., Manoussakis G. and Nikolov G. St., *Inorg. Chim. Acta*, **195**, 227 (1992).
[25] Christidis P., Triantafyllou S. T. and Bolos C., *Z. Kristall.*, **209**, 502 (1994).
[26] Boggs R. and Donohue J., *Acta Cryst.*, **B31** (1975).
[27] Templeton D. M. and Sarker B., *Canad. J. Chem.*, **63**, 3112 (1985).
[28] *QCMP113, Version 6.0* by Koch R. and Wiedel B., Bloomington, Indiana, USA.
[29] *QCMP116, ICONC program* by Calzaferri G. and Braendle M., Bloomington, Indiana, USA.
[30] Fitzpatrick, N.J. and Murphy G.H., *Inorg. Chim. Acta*, **87**, 41 (1984); **111**, 139 (1986).
[31] Christidis P., Bolos C. and Nikolov G. St., *Inorg Chim Acta*, **237**, 123 (1995).
[32] Geary W. J., *Coord. Chem. Rev.*, 7, 81 (1971).
[33] (a) Carter G. R. in: *Diagnostic Procedures in Veterinary Bacteriology and Mycology* 3rd edn., Charles C. Thomas Publ., Springfield, IL., p. 347 (1979). (b) Stokes J. E. in: *Clinical Bacteriology*, 4th edn., Edward Arnold, Great Britain, p. 208 (1975)
[34] Atkins G. J., Johnson M. D., Westmacott L.M. and Burke D. C., *J. Gen. Virol.*, **25**, 381 (1974)
[35] Wilkinson G., Gillard R. D. and McCleverty J. A., eds. in: *Comprehensive Coordination Chemistry*. Vol. **2** (1987). *Ligands;* Vol. **5**. (1987) *Late Transition Elements*. Pergamon Press, Oxford.

**Received: October 7, 1998 - Accepted: October 20, 1998 - Received in revised camera-ready format: October 23, 1998**

